# Fallopian tube vascularization observed by microfocus computed tomography

**DOI:** 10.1590/0100-3984.2018.0120

**Published:** 2020

**Authors:** Pedro Teixeira Castro, Osvaldo Luiz Aranda, Haimon Diniz Lopes Alves, Ricardo Tadeu Lopes, Heron Werner, Edward Araujo Júnior

**Affiliations:** 1 Radiology Department, Universidade Federal do Rio de Janeiro (UFRJ), Rio de Janeiro, RJ, Brazil.; 2 Department of Obstetrics and Gynecology, Hospital Universitário Severino Sombra, Vassouras, RJ, Brazil.; 3 Nuclear Engineering Program (PEN/COPPE), Universidade Federal do Rio de Janeiro (UFRJ), Rio de Janeiro, RJ, Brazil.; 4 Radiology Department, Clínica de Diagnóstico Por Imagem (CDPI), Rio de Janeiro, RJ, Brazil.; 5 Department of Obstetrics, Escola Paulista de Medicina da Universidade Federal de São Paulo (EPM-Unifesp), São Paulo, SP, Brazil.; 6 Medical Course, Universidade Municipal de São Caetano do Sul (USCS), Campus Bela Vista, São Paulo, SP, Brazil.

## INTRODUCTION

In addition to its fundamental role in human fertilization, the fallopian tube has recently been associated with the risk of epithelial ovarian cancer, the most aggressive and deadly gynecological malignancy. Substantial evidence, based on combined morphological and molecular data, indicates that the distal fallopian tube (fimbria), rather than the ovarian surface epithelium, is the location of origin of most low- and high-grade serous ovarian carcinomas^1,2^. The tubal fimbria and ovarian epithelium have similar histological characteristics and immunohistochemical markers, supporting the hypothesis that, embryologically, the fimbria arise separately from the rest of the fallopian tube^3^.

Microfocus computed tomography (micro-CT) is a non-destructive X-ray technology that provides high-resolution three-dimensional (3D) images of *ex vivo* specimens. Given its 3D capability, micro-CT enables the differentiation of contrast-enhanced tissues and structures. It also plays an important role in the study of vascular development and the mechanism of neovascularization in animals^4-7^. Here, we report the micro-CT results of fallopian tube studies to improve understanding of its structure and architecture.

## PROCEDURE

Samples used in this study were obtained from the disease-free fallopian tubes of two women of reproductive age (38 and 43 years, respectively) who underwent total abdominal hysterectomy and salpingectomy for benign conditions at the Severino Sombra University Hospital, in the city of Vassouras, Brazil. The uterine segments of the fallopian tube samples were excluded. The local research ethics committee approved this study (Reference No. 56031916.0.0000.5290), and both of the patients involved gave written informed consent.

The two samples were fixed in 10% formalin for more than 24 h at room temperature before iodine staining for micro-CT imaging. After the samples had been washed twice with distilled water, they were immersed in 10% Lugol’s solution for 24 h. The specimens were then removed from the staining solution and rinsed with phosphate-buffered saline to remove excess stain and prevent surface saturation. Images were then acquired in a micro-CT scanner (SkyScan 1173 v.1.6.9.4; Bruker microCT, Kontich, Belgium) at 40 kV and 200 µA, with a pixel size of 34.1 µm, after which the samples were returned to 10% formalin for destaining and histological analysis. Morphological analysis and 3D reconstruction were performed on reconstructed images using the CTan software, version 1.16 (Bruker micro-CT), after manual selection of connected, radiopaque voxels and the tubal lumen.

**Figure 1:**
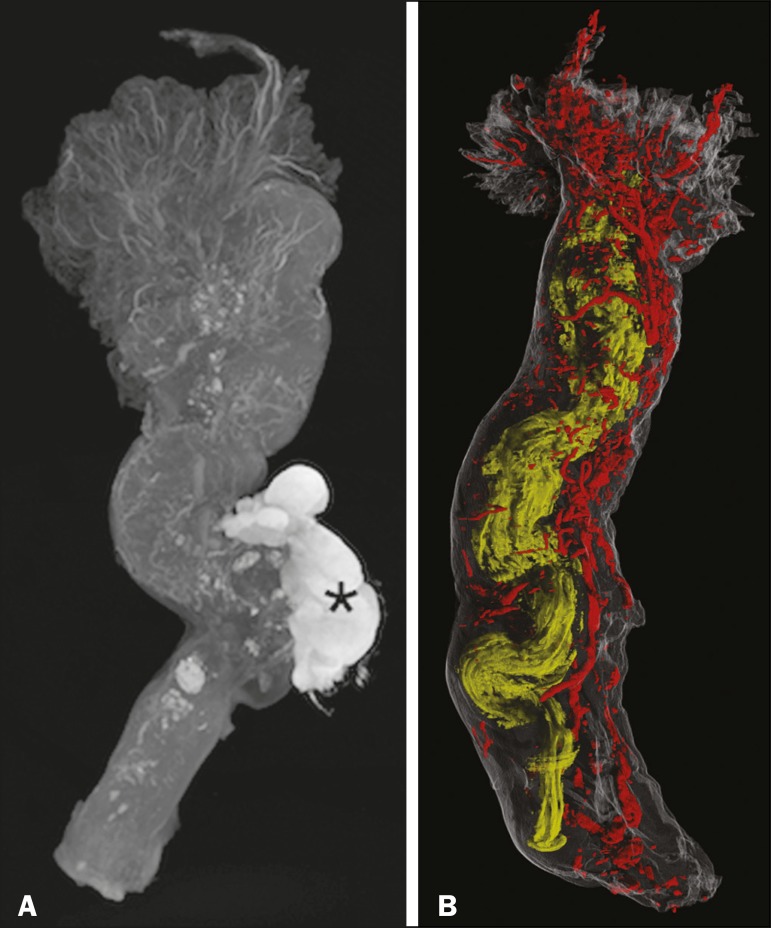
**A:** Micro-CT image of the fallopian tube. The vessels are less radiolucent, showing contrast with the tissue and the abundant blood vessels in the fimbria, especially at their ends. Wolffian duct remnants (asterisk). **B:** Micro-CT reconstruction of the fallopian tube. The tortuosity, diameter, and folding of the lumen (in yellow) increases toward the fimbria. The vessels (in red) follow the external contour of the fallopian tube, their volume increasing in the fimbria.

Along the rectilinear fallopian tube, the vessels followed the tubal serosa. In the lumen and thick muscle portion of the isthmus, the vessels followed the mucosa (Figure 1A). After the transition to and along the ampulla, the mucosa became extremely folded and tortuous, although the linear pattern of the vessels was sustained until the transition to the fimbria. The vascular network of the fimbria was abundant and robust compared with that observed in other segments of the fallopian tube. The vessels were abundant, branching to the ends of the fimbria (Figure 1B).

In summary, micro-CT showed vascularization of the fimbria disproportional to that of the rest of the fallopian tube. Micro-CT allows detailed observation of the microanatomy of human tissue and can be helpful in studies of soft tissues. Further studies are needed in order to confirm this finding and to investigate the relationship between this abundant vascularization and physiological conditions of the fallopian tube.
